# Correlation of red blood cell parameters and platelet count among adult anemic patients attending Madda Walabu University Goba Referral Hospital, Goba, Southeast Ethiopia: A comparative cross-sectional study

**DOI:** 10.1097/MD.0000000000041156

**Published:** 2025-01-10

**Authors:** Mohammedamin Jundi, Edosa Tadasa, Wondimagegn Adissu

**Affiliations:** a School of Medical Laboratory Sciences, Faculty of Health Sciences, Madda Walabu University, Goba, Ethiopia; b School of Medical Laboratory Sciences, Faculty of Health Sciences, Institute of Health, Jimma University, Jimma, Ethiopia; c Clinical Trial Unit, Jimma University, Jimma, Ethiopia.

**Keywords:** anemia, bale, correlation, ethiopia, platelet count, red cell parameter

## Abstract

Anemia is a worldwide public health problem and is associated with platelet disorders. The relationship between anemia and platelets is complex, with the association being either normal platelet count or thrombocytosis. Platelets are significantly decreased in patients with anemia, and thrombocytopenia has been documented in patients with severe anemia. There are few reports in the literature on the correlation between platelet count and red blood cell parameters in anemic patients. Therefore, this study aimed to compare the correlation between red blood cell parameters and platelet count in adult anemic patients attending Madda Walabu University Goba Referral Hospital (MWUGRH): Goba, Southeast Ethiopia, from May 30 to July 30, 2022. A cross-sectional comparative study was conducted among 352 subjects (176 anemic and 176 controls) who attended the hospital during the study period and were recruited using a convenient sampling technique. Sociodemographic and other relevant variables were collected using a structured questionnaire. Four milliliters of venous blood were collected and placed in a K_2_EDTA tube for analysis of hematologic parameters using the Sysmex XN-550 (Sysmex Corp., Japan) automated hematology analyzer; the hemoglobin value was used to determine anemia status. Data were entered into Epidata Manager, version 4.6.0.2, and analyzed using SPSS Statistical Software, version 25. The independent-sample *t*-test was used to compare parameters between groups, and correlation statistics was used to correlate parameters between groups. Chi-square was used at a 95% confidence interval, considering *P* < .05 statistically significant for association among categorical variables. Correlation analysis showed that platelet count was significantly, positively correlated with RBC, and negatively correlated with MCV, MCH, and MCHC (*r* (*P*) = 0.168 (.026) and −0.252 (.000), −0.275 (.001), −0.218 (.004), respectively). It was also negatively correlated with HGB and HCT levels in the healthy control (*r* (*P*) = −0.266 (.000) and −0.149 (.049) respectively). Morphologically, 44.32% were microcytic hypochromic anemia, 53.98% were normocytic normochromic anemia and 1.70% were macrocytic anemia. The findings showed that platelet count variation correlates well with red cell indices and morphologic types of anemia in adult anemic patients. Such correlation will enable physicians to make diagnoses and administer treatments.

## 1. Introduction

Anemia is a condition in which the number of red blood cells (RBC) or the concentration of hemoglobin (Hgb) in them is lower than normal, resulting in insufficient oxygen-carrying capacity to meet the body’s physiological needs; these physiological needs vary according to age, sex, altitude, pregnancy, and stage of the disease.^[1,2]^ History, physical examination, signs and symptoms, Hgb values, and other procedures and findings are used to make a clinical diagnosis.^[3,4]^

The causes of anemia throughout the world are multifactorial.^[5]^ It is estimated that half of the world’s anemia cases are due to iron deficiency (ID); however, these reports did not assess the relative contribution of ID and other risk factors at the regional or country level.^[6]^ Another cause may include parasitic diseases and infections such as malaria, hookworm, and schistosomiasis; other micronutrient deficiencies such as folic acid, vitamin A, and vitamin B12; and genetic hemoglobinopathies such as sickle cell disease and thalassemia.^[7]^

Anemia can be classified into microcytic hypochromic, normocytic normochromic, and macrocytic anemia based on the morphologic criteria of RBC in the thin blood film or on red cell indices- mean cell volume (MCV), mean cell hemoglobin (MCH), and mean cell hemoglobin concentration (MCHC).^[8]^ On the other hand, based on Hgb concentration, its severity can be categorized as mild, moderate, and severe anemia.^[1]^

Anemia was defined by the World Health Organization (WHO) criteria as a hemoglobin concentration of <12.0 g/dL in women and <13.0 g/dL in men.^[9]^ Women with Hgb values between 11 and 12 g/dL, 8 to 11 g/dL, and < 8 g/dL were categorized as having mild, moderate, and severe anemia, respectively. In men, anemia < 13.0 g/dL, mild 11 to 12 g/dL, moderate 8 to 11 g/dL, and severe < 8 g/dL were classified as the severity of anemia.^[10,11]^

Platelets are nonnucleated and round. Their diameter is between 2 and 4 micrometers. The function of platelets is to help in homeostasis. Their typical number ranges from 150 × 10^9^/L to 450 × 10^9^/L. They develop in the bone marrow. They play a role in the formation of platelet plugs, retraction of blood clots, and healing of blood vessels.^[12]^ Mature RBCs and PLTs have similar physiological rhythms and are simultaneously involved in numerous pathological conditions.^[13]^ Both RBCs and PLT have a stage in the peripheral blood called reticulocytes for RBCs and reticulated PLT for PLT.^[14]^ The cytokine growth factor erythropoietin (EPO) has a significant effect on both cell series.^[15]^ All available hematology analyzers calculate the RBC volume and PLTs using the same aperture and dilution.^[14]^

The relationship between iron deficiency anemia (IDA) and PLT is complex; ID is usually associated with either a normal PLT count or thrombocytosis.^[16]^ In rare cases, some patients develop transient neutropenia after the correction of IDA.^[17]^ In rare cases, IDA may be associated with thrombocytopenia, and when IDA is corrected, thrombocytopenia improves concomitantly.^[18]^

Previous studies have shown that PLT is significantly lower in patients with anemia than in control subjects.^[18]^ They reported thrombocytopenia in patients with severe anemia when Hgb is < 7 g/dL.^[1]^ The cause of thrombocytopenia in severe anemia could be due to a high endogenous erythropoietin response.^[19]^ In severe IDA, the number of megakaryocytes decreases whereas the size of megakaryocytes increases.^[14]^ This may be due to the shortening of megakaryocyte maturation and a decreased influx of precursors.^[16]^ The mechanism of thrombocytopenia in ID could be an early response to direct stimulation of the EPO receptor on the megakaryocyte or a detour into the erythroid progenitor cell pathway, resulting in decreased platelet formation.^[20]^

With the remarkable advances in technology and the advent of fully automated analyzers, it is easy to analyze various parameters of RBCs and PLTs, such as erythrocyte count, hematocrit (Hct), MCV, RDW, PLT count, platelet crit (PCT), mean platelet volume (MPV), and platelet distribution width (PDW).^[21]^ These parameters are often under-reported due to a lack of validated knowledge about their clinical utility.^[13]^

The linear relationships between various red blood cell parameters (red blood cell count, Hct, MCV, RDW, HGB) and PLT parameters (PLT count, PCT, MPV, PDW) in any given physiological or pathological condition were determined and their predictive value in certain pathological conditions (anemia) was determined.^[22]^ Since anemia is a symptom of underlying diseases, the various RBC and PLT parameters and their variations can be further investigated to determine if there is a relationship between them.^[23]^

However, there is little information on the relationship between low and high RBC parameters and PLT count. There are few reports in the literature on the correlation between PLT and RBC parameters (indices). Such a study is important because many anemic patients are associated with platelet disorders. This study aimed to assess the correlation between RBC parameters and PLT count in adult anemic patients attending Madda Walabu University Goba Referral Hospital in Bale Zone, Ethiopia.

## 2. Materials and methods

### 2.1. Study area, design, and period

This study was conducted at Madda Walabu University Goba Referral Hospital (MWUGRH). The hospital is located in Goba town, located 445 km southeast of Addis Ababa, the capital city of Ethiopia, at an average elevation of 2700 meters.^[24]^ A comparative cross-sectional study was conducted from May 30 to July 30, 2022.

### 2.2. Study participants

All adult patients requiring complete hematological investigation in MWUGRH. Non-anemic and healthy blood donors attending Goba Blood Bank, staff of Goba Hospital, and students of MWUGRH during the study period.

#### 2.2.1. Eligibility criteria

All anemic patients who were more than or equal to 15 years of age were included in the study based on their Hgb levels. Patient recruitment was done consecutively without prior effect on the recruitment to make the participation random from the outpatient department. An apparently healthy study participant of the same age group was included after screening by staff and regular voluntary blood donors as control. Patients with acute hemorrhage, infection, malignancy, and chronic inflammatory diseases were excluded from the study as this may cause reactive thrombocytosis. Besides, infants, young children, and pregnant women have variable complete cell counts and other hematological values due to physiological and other conditions.

### 2.3. Sample size determination and sampling technique

The sample size was calculated based on 2 population mean formulas using G*-Power statistical free software version 3.1, by considering the following assumptions: 95% confidence level (2- tailed, α = 0.05), 80% power (β = 0.20), the ratio of sample size (anemic/control) was 1:1, effect size (d) was 0.3125, and 10% nonrespondent rate.^[25]^ The mean and standard deviation (SD) [mean ± SD] of Hgb (g/dL) for the anemic and CGs were taken from a study conducted in Saudi Arabia^[26]^ 9.64 ± 1.13 for the anemic group and 14.06 ± 1.39 for the CG. The sample size was determined to be 176 for each group, and 352 study participants were included in this study. Study participants were recreated using a convenient sampling technique.

### 2.4. Data collection methods

Data on sociodemographic characteristics were collected using a structured questionnaire. The questionnaire was adapted from other similar studies^[27]^ and was prepared in English. It was translated into the local language. Finally, it was translated back into English to ensure consistency. Before data collection commenced, all data collectors underwent comprehensive training provided by the principal investigator that covered: the administration of the structured questionnaire, ethical considerations, including informed consent and participant interaction, about maintaining confidentiality. Informed consent was obtained from the study participants which included a detailed explanation of the study’s purpose, procedures, risks, and benefits provided in the local language before data collection. To ensure data confidentiality, the study participants were identified using codes instead of individual identifiers, and unauthorized persons were not able to access the collected data. After an interview, a review of records, signs or symptoms of diseases, and measurement of vital signs were completed by trained clinical nurses, and the study subjects were sent to a laboratory where a blood sample was collected for determination of hematological parameters.

About 4 mL of venous blood sample was collected in ethylene di-amine tetra acetic acid (K_2_EDTA) tubes by laboratory technologists from each study participant who suspected anemia case was requested for hematological parameter analysis and blood film preparation. On the other hand, 4 mL milliliters of venous blood was collected into an EDTA test tube from the CG (blood donor) at the time of donation. All samples received in the laboratory were checked for quality and labeled by code numbers. Hematological parameters like; Hgb, RBC, Hct, MCV, MCH, MCHC, and RDW were determined using the automated blood analyzer Sysmex XN-550 (Sysmex Corp,Kobe, Hyogo, Japan). Thin blood films were prepared, air-dried, labeled, and then stained with Wright stain to evaluate the morphological classification of anemia in anemic study participants for confirmatory to CBC.

A control group (CG) comprised healthy individuals with no clinical signs or symptoms of disease, including acute inflammatory/infectious conditions, normal hematologic findings, and C-RP < 5 mg/L. Healthy individuals were students or laboratory staff, all of whom voluntarily donated blood samples. In this study, Anemia was defined as a level of Hgb < 13 g/dL for males and < 12 g/dL for nonpregnant women,^[1]^ Thrombocytosis was defined as a High PLT count above the normal range (PLT count > 450 × 10^9^/L), and thrombocytopenia was defined as a low PLT count below the normal range (PLT count < 150 × 10^9^/L).

### 2.5. Data quality assurance

This study used quality assurance procedures to ensure that the data were valid and reliable. To ensure data quality, data collectors received training and orientation before data collection. Furthermore, the questionnaire was translated into Amharic and Afan Oromo, then back into English, and pretested on a 5% sample at Goba Hospital to guarantee clarity and accuracy.

Standard operating procedures (SOPs) were developed and implemented for pre-analytical, analytical, and post-analytical procedures. Blood specimens were thoroughly checked, and any specimens with hemolysis, clotting, inadequate volume, or incorrect labeling were excluded. To achieve accurate results with the Sysmex XN-500 hematological analyzer, commercially available quality controls (high, medium, and low) were used daily during startup. Randomly selected specimens were subjected to repeated analysis to determine instrument performance and reproducibility (delta check).

Daily routine maintenance of equipment was performed following the manufacturer’s instructions to check for background issues, vacuum faults, adequate cleaning, and suitable start-up and shutdown procedures. All laboratory examination findings were carefully documented in a standardized report format and were attached to the appropriate questionnaire with a unique identification number. In circumstances where the analyzer raised flags or to validate a complete blood count (CBC), a blood film examination was performed to assess the morphological classification in anemic individuals. Regular checks were performed to ensure that the acquired data were complete, clear, and accurate, after which they were correctly transcribed and checked.

### 2.6. Data processing and analysis

The data was input into EpiData version 4.6.02 (EpiData Association, Odense, Denmark) after being verified for consistency and completeness. The statistical package for social sciences (SPSS) version 25 software (IBM Corporation, Armonk, NY) was then used to import and analyze the data. A histogram, the Kolmogorov–Smirnov test, and the Shapiro–Wilk test were used to evaluate the normality of the data distribution. For categorical variables, the results were presented as percentages and frequencies, and for continuously and normally distributed variables, the mean ± SD was used. For categorical data, statistical differences between groups were determined using the chi-square test. The independent t-test was used for normally distributed data, and the Mann–Whitney *U* test was used for non-normally distributed data to compare the hematological parameters between the anemic and control individuals. To evaluate the relationship between hematological parameters and independent variables, normally distributed data were correlated using Pearson correlation, whereas non-normally distributed data were correlated using Spearman correlation. A ONE-WAY ANOVA was performed on continuous dependent variables. The WHO recommended that hemoglobin (Hgb) readings be corrected using a factor of 1.3 at an average altitude of 2700 meters to diagnose anemia.^[1]^ A *P*-value < .05 was used to assess statistical significance. Tables and figures are used to display the results.

## 3. Results

### 3.1. Sociodemographic characteristics of study participants

A total of 352 study participants (176 anemic patients and 176 healthy controls) were included in this study. Most of the study participants 111 (63.1%) were females for anemic patients and 90 (51.1%) were males for healthy controls. The mean age (mean ± SD) was 28.32 ± 9.3 for anemic patients and 28.88 ± 8.93 years for healthy controls. Out of the total study participants, approximately 59 (33.5%) were able to read and write for anemic patients and 128 (72.7%) had higher educational levels for healthy controls. About 112 (63.6%) and 156 (88.8%) were from urban residences for anemic patients and healthy controls, respectively (Table 1).

**Table 1:** Sociodemographic characteristics of study participants attending MWUGRH; Goba; Southeast Ethiopia, May 30 to July 30, 2022 (n = 352).

Variables	Anemic patients n (%)	Healthy control n (%)	Total n (%)
Age in years			
15 to 30	105 (59.6)	125 (71.0)	230 (65.3)
31 to 45	33 (18.8)	38 (21.6)	71 (20.2)
46 to 60	18 (10.2)	13 (7.4)	31 (8.8)
61 to 85	20 (11.4)		20 (5.7)
Gender			
Female	111 (63.1)	86 (48.9)	197 (55.9)
Male	65 (36.9)	90 (51.1)	155 (44.1)
Residence			
Urban	112 (63.6)	156 (88.6)	268 (76.14)
Rural	64 (36.4)	20 (11.4)	84 (23.86)
Educational status			
Unable to read and write	46 (26.1)	4 (2.3)	50 (14.4)
Able to read and write	59 (33.5)	38 (21.6)	97 (27.5)
Primary school	35 (19.9)	5 (2.8)	40 (11.3)
Secondary school	18 (10.2)	1 (0.6)	19 (5.3)
College/University	18 (10.2)	128 (72.7)	146 (41.4)
Occupational status			
Farmer	19 (10.7)		19 (5.4)
Daily labor	39 (22.1)	24 (13.6)	63 (17.8)
Governmental/employee	23 (13.1)	27 (15.3)	50 (14.2)
Student	39 (22.1)	94 (53.4)	133 (37.7)
Housewife	44 (25.0)	18 (10.2)	62 (17.6)
Merchant	12 (6.8)	13 (7.4)	25 (7.1)
Marital status			
Single	56 (31.8)	100 (56.8)	156 (44.3)
Married	108 (61.3)	76 (43.2)	184 (52.2)
Divorced	7 (3.9)		7 (1.9)
Widowed	5 (2.8)		5 (1.4)
Monthly Income			
Below 500	67 (38.1)	13 (7.4)	80 (22.7)
600 to 5000	57 (32.4)	101 (57.4)	158 (44.8)
5050 to 10,000	18 (10.2)	29 (16.5)	47 (13.3)
Above 10,000	15 (8.5)	19 (10.8)	34 (9.6)
Family dependent	19 (10.8)	14 (8.0)	33 (9.3)

Note: n – frequency.

Abbreviation: MWUGRH = Madda Walabu University Goba Referral Hospital.

### 3.2. Hematological (RBC and PLT parameters) indices

The finding showed that anemic patients exhibited significantly higher PLT count (313.16 ± 139.26 vs 267 ± 68.38), RDW (15.59 ± 3.70 vs 12.81 ± 0.95), and PCT (0.30 ± 0.12 vs 0.26 ± 0.06). Also, anemic patients had significantly lower RBC (4.21 ± 0.86 vs 5.14 ± 0.41), Hgb (9.81 ± 2.05 vs 14.85 ± 1.02), and Hct (33.32 ± 6.70 vs 44.39 ± 3.94) compared to controls (Table 2).

**Table 2:** Selected hematological indices of study participants attending MWUGRH; Goba; Southeast Ethiopia, May 30 to July 30, 2022 (n = 352).

RBC and Platelet parameters	Anemic patients			Healthy control			*P*-value
	(mean ± SD)	Min	Max	(mean ± SD)	Min	Max	
Hgb (g/dL)	9.81 ± 2.05	3.30	12.80	14.85 ± 1.02	12.70	17.10	.001
Hct (%)	33.32 ± 6.70	9.20	43.20	44.39 ± 3.94	37.90	54.80	.001
RBC (×10^12^/L)	4.21 ± 0.86	0.88	6.19	5.14 ± 0.41	3.87	6.23	.001
MCV (fL)	79.99 ± 10.15	50.70	118.40	86.83 ± 4.38	76.70	100.80	.001
MCH (pg)	26.17 ± 4.38	12.10	41.20	28.79 ± 2.67	24.70	34.90	.001
MCHC (g/dL)	32.56 ± 2.27	23.70	42.20	33.36 ± 1.22	30.00	41.00	.001
RDW (% CV)	15.59 ± 3.70	11.00	35.70	12..81 ± 0.95	10.90	19.40	.001
PLT (×10^9^/L)	313.16 ± 139.26	12.00	996.00	267 ± 68.38	121.00	474.00	.001
PDW (fL)	10.59 ± 2.23	7.30	23.90	11.02 ± 1.67	7.60	18.20	.042
MPV (fL)	9.81 ± 1.03	7.00	14.70	9.98 ± 0.80	8.40	11.90	.076
PCT (%)	0.30 ± 0.12	0.01	0.87	0.26 ± 0.06	0.13	0.47	.001

Note: The *P* < .05 is statistically significant in independent *t*-test,

Abbreviations: dL = deciliter, fL = femtoliter, g/dL = gram per deciliter, HCT = hematocrit, HGB = hemoglobin, L = liter, Max = maximum, MCH = mean cell hemoglobin, MCV = mean cell volume, Min = minimum, MPV = mean platelet volume, PDW = platelet distribution width, pg = picogram, RBC = red blood cell, RDW = red cell distribution width, SD = standard deviation.

### 3.3. RBC parameters and PLT count variation

There were significant differences in MCV, MCH, MCHC, RBC, Hgb, Hct, and RDW across the 3 PLT count variations. There was a significant decrease in Hgb and RDW in the normal count and thrombocytosis compared to thrombocytopenia. Also, there was a significant decrease in RBC count and Hct in thrombocytosis compared with thrombocytopenia and normal count. The MCV, MCH, and MCHC values were significantly increased in thrombocytosis compared with thrombocytopenia and normal count (Table 3).

**Table 3:** Selected hematological indices over the 3 PLT count variation in adult anemic patients’ (Mean ± SD) attending MWUGRH; Goba; Southeast Ethiopia, May 30 to July 30, 2022 (n = 352).

RBC and its indices	Thrombocytopenia	Normal count	Thrombocytosis	*P*-value		
				1 and 2	1 and 3	2 and 3
RBC count (×10^12^/L)	2.86 ± 1.27	4.30 ± 0.73	4.29 ± 0.88	.001	.001	.999
HGB (g/dL)	7.01 ± 2.37	10.15 ± 1.84	8.95 ± 2.16	.001	.018	.017
HCT (%)	22.86 ± 9.13	34.38 ± 5.95	31.64 ± 5.36	.001	.001	.127
MCV (fL)	82.90 ± 10.88	80.45 ± 9.35	75.58 ± 13.62	.717	.123	.090
MCH (pg)	27.71 ± 5.48	26.46 ± 2.16	23.55 ± 4.44	.619	.025	.009
MCHC (%)	33.19 ± 2.60	32.76 ± 2.22	31.12 ± 1.95	.812	033	.004
RDW (% CV)	18.87 ± 6.26	15.12 ± 3.28	17.06 ± 3.61	.003	.358	.048

Note: The *P*-value is derived from ANOVA, 1st: thrombocytopenia, 2nd: normal count, 3rd: thrombocytosis.

Abbreviations: fL = femtoliter, HCT = hematocrit, HGB = hemoglobin, MCH = mean cell hemoglobin, MCHC = mean cell hemoglobin concentration, MCV = mean cell volume, pg = picogram, RBC = red blood cell count, RDW = red cell distribution width.

### 3.4. Correlations between PLT count and RBC parameters

The correlation analysis showed that PLT count was significantly, positively correlated with RBC, and negatively correlated with MCV, MCH, and MCHC (*r* (*P*) = 0.168 (.026) and −0.252 (.001), −0.275 (.001), −0.218 (.004) respectively) in anemic patients. It was also negatively correlated with Hgb and Hct levels in the healthy controls (*r* (*P*) = −0.266 (.001) and −0.149 (.049) respectively), and positively correlated with RDW (Table 4).

**Table 4:** Pearson correlations (*r* (*P*)) between RBC indices and PLT count in the study participants attending MWUGRH, Goba, Southeast Ethiopia, May 30 to July 30, 2022 (n = 352).

RBC and its indices	Anemic patients	Healthy control
	Platelet count (×10^9^/L)	Platelet count (×10^9^/L)
	*r* (*P*)	*r* (*P*)
RBC count (×10^12^/L)	0.168* (.026)	−0.096
Hb (g/dL)	−0.052	−0.266* (.001)
HCT (%)	0.009	−0.149* (.049)
MCV (fL)	−0.252* (.001)	−0.122
MCH (pg)	−0.275* (.001)	−0.101
MCHC (%)	−0.218* (.004)	−0.139
RDW (% CV)	0.085	0.152* (.001)

Abbreviations: HCT = hematocrit, HGB = hemoglobin, MCH = mean cell hemoglobin, MCHC = mean cell hemoglobin concentration, MCV = mean cell volume, RBC = red blood cell, RDW = red cell distribution width.

* Significant correlation.

### 3.5. Morphological types of anemia in adult anemic patients

**Figure 1.**
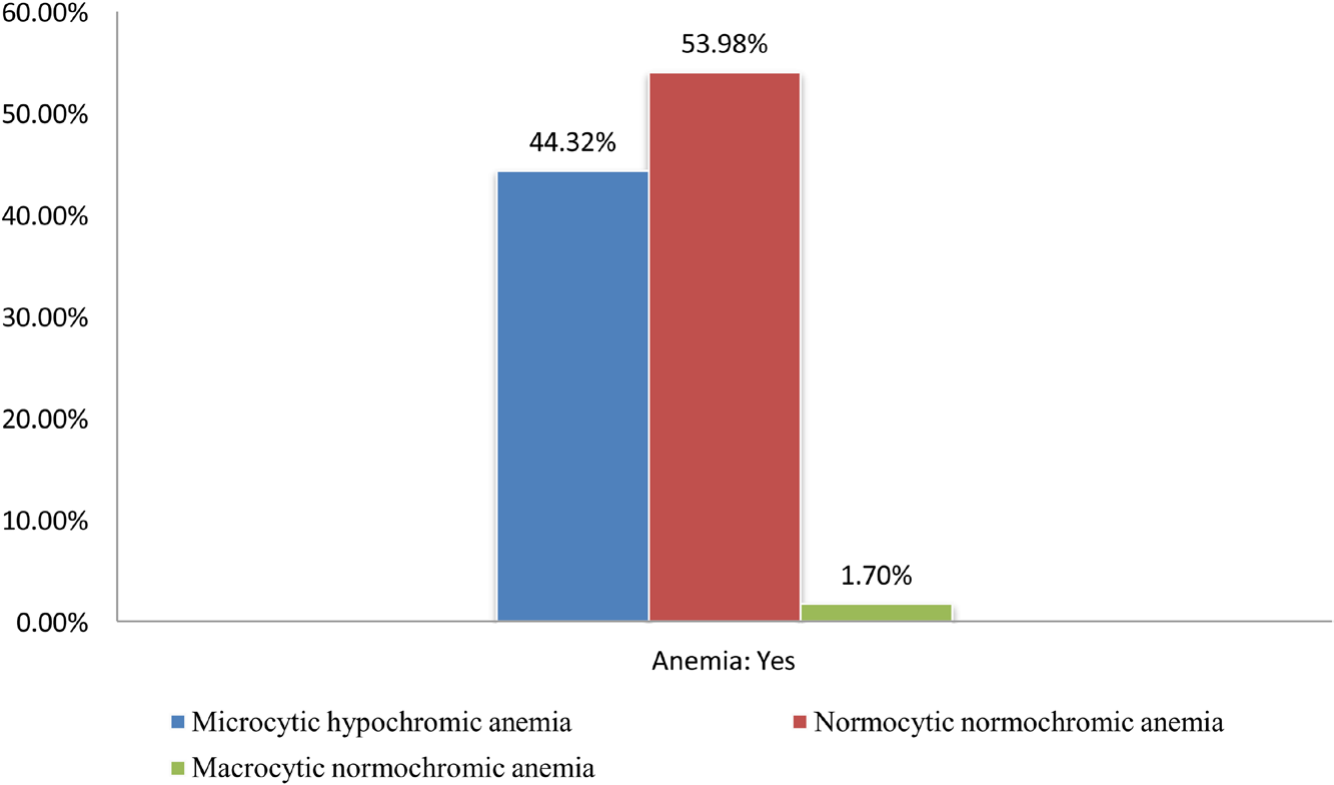
Morphological types of anemia among adult anemic patients attending MMWUGRH; Goba; Southeast Ethiopia, May 30 to July 30, 2022 (n = 352). MWUGRH = Madda Walabu University Goba Referral Hospital, PLT = platelet.

Normocytic normochromic anemia was the most common type, followed by microcytic hypochromic and macrocytic anemia (Fig. 1).

### 3.6. Platelet count variation among adult anemic patients

**Figure 2.**
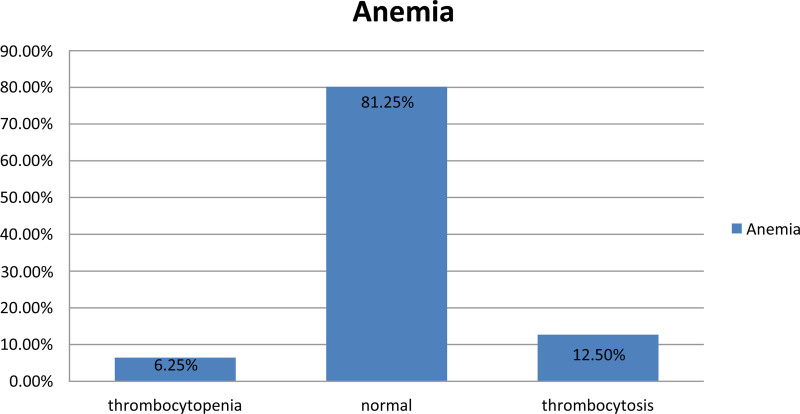
Total PLT count variation in adult anemic patients attending MWUGRH; Goba; Southeast Ethiopia, May 30 to July 30, 2022 (n = 352). MWUGRH = Madda Walabu University Goba Referral Hospital, PLT = platelet.

Similarly, total PLT count variation was calculated using an automated blood cell Sysmex machine (counter-checked manually via peripheral blood smear findings for the flagged ones). The majority revealed normal counts followed by thrombocytosis and thrombocytopenia in anemic patients (Fig. 2).

**Figure 3.**
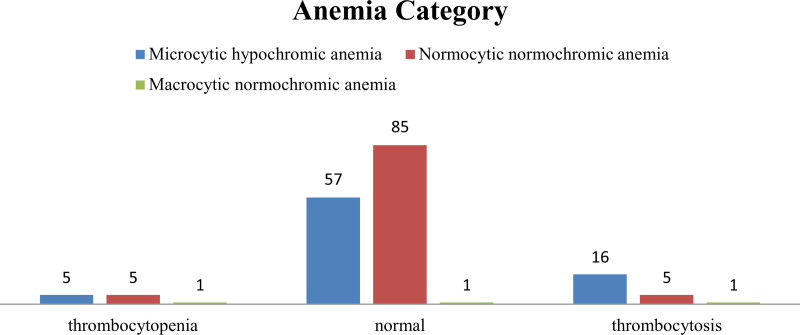
PLT count variation was cross-tabulated with morphological types of anemia in adult anemic patients attending MWUGRH; Goba; Southeast Ethiopia, May 30 to July 30, 2022 (n = 352). MWUGRH = Madda Walabu University Goba Referral Hospital, PLT = platelet.

The morphological types of anemia were cross-tabulated with the PLT count variation obtained in the study (using the chi-square test), revealing a positive association between them and statistical significance (*P*-value = .005) (Fig. 3).

## 4. Discussions

Anemia continues to be a serious global public health issue with negative effects on both social and economic development and human health.^[28]^ Adults with anemia are more likely to experience unfavorable effects, including decreased work productivity, greater susceptibility to various illnesses, increased hospitalization, and increased mortality.^[29]^

In this study, there was a statistically significant increment in RDW, and PCT of adult anemic patients compared with the control. The mean PLT count was also higher in adult anemic patients than in the control. This finding was in coherence with a report of studies conducted in Austria.^[30]^ A possible explanation for the increased PLT count might be that it arises from enhanced production, release from the spleen, or prolonged platelet survival. The number of platelets can also increase due to the division of platelets in the blood circulation.^[31]^ Because of persistent thrombocytosis in our model, the increase in the number of platelets is not possible due to splenic release or long half-life, but rather to the increase in production.^[30,32,33]^ In contrast, in the study conducted in Turkey,^[34]^ the mean PLT count was lower as compared to the control. A possible explanation might be that the study was retrospectively conducted for more than 2 years with a large sample size, and many severe anemia cases with ID were obtained. Another study was conducted in Tennessee, USA^[35]^ and in contrast to this study, lower mean PLT was documented. The reason behind this might be that the study was conducted on children below 18 years.

The current study found that the PLT count of anemic patients was strongly connected with RBC count and negatively correlated with MCV, MCH, and MCHC when compared with the control. The finding was consistent with that of previous Indian study,^[36]^ 1 of which, MCV, had a statistically significant inverse relationship (*R* = −0.224, *P*-value = .006) with the PLT count. However, the association was statistically insignificant, with a *P*-value of .197.^[37]^

Similarly, the RBC count in this study was relatively strongly correlated with the PLT count, which was statistically significant (*r* = 0.168, *P*-value = .026) in anemic patients. This significant correlative finding thus reflects the directly proportional relationship between the PLT count and RBC values in anemic patients, reflecting the relationship between EPO and thrombopoietin (TPO).^[23]^ The possible reason behind this is that EPO, the regulator of erythropoiesis, has structural similarity with TPO, the stimulator of megakaryopoiesis. Increased EPO and its structural analogy to TPO have been considered a possible mechanism for the correlation of platelet count with RBC count in anemic patients.^[33]^

In the current study, we observed that Hgb as an index of anemia was significantly and weakly negatively correlated with platelet count among adult anemic patients compared with controls. This finding was supported by previous studies conducted in India,^[23,36]^ which reported that PLT count was significantly negatively correlated with Hgb in anemic patients compared with the CG. Similar kinds of correlative findings between PLT count and Hgb concentration level were also found in the study done in India.^[38]^

In contrast, in a study conducted in India,^[37,39]^ the Hgb concentration level of the study population had a positive correlation with the platelet count compared with the control. This indicates the minor effects between these 2 parameters. The reason for the relationship between low and high platelet counts on hemoglobin levels may be related to the normal physiology of hematopoiesis, where all blood components originate in the bone marrow. Similarly, there was a statistically significant positive association between platelet count and RBC count. Hence, if the bone marrow is affected, it would affect all of the blood cells, including Hgb and PLT concentration levels.^[37]^

Based on the morphological classification of anemia, normocytic normochromic anemia (53.98%) and microcytic hypochromic anemia (44.32%) were the 2 most common types among adult anemic individuals. This finding was consistent with studies conducted in Italy and Brazil that showed 88% normocytic and 72.3% cases of microcytic anemia, respectively.^[40,41]^ Another study reported a 46% prevalence of normocytic normochromic anemia as the most common type of anemia.^[42]^ On the contrary, other studies reported that the vast majority of anemia cases were microcytic, which is the second most prevalent anemia in this study.^[11,43]^

On the other hand, the platelet count variation in this study revealed predominantly a normal count of 143 (81.25%) cases, followed by thrombocytosis and thrombocytopenia. Unlike this study’s findings, a study done in India^[39]^ revealed thrombocytopenia as the most common outcome within total platelet count variation. Likewise, thrombocytopenia and thrombocytosis were also observed in normocytic and microcytic hypochromic anemia as 5 (5.5%) and 16 (3.5%), respectively, in this study. Such variation in platelet count among anemic patients may be related to erythropoietin (EPO) and thrombopoietin (TPO), as mentioned above.

Mild anemia was the most common type in this study, and moderate anemia is more common than severe anemia. This finding was similar to a study from Pakistan, which reported that most cases of anemia observed were mild in severity.^[44]^ The age groups with the highest rate of anemia (59.7%) were those between 15 and 30 years old, whereas those between 61 and 85 years old had the lowest rate of anemia (11.4%). The average rate of anemia in the elderly has been reported in other studies to be 11.8%, 5.4%, and 8 to 44%, which is comparable to our findings.^[45]^

Other studies have also reported that elderly people’s anemia may be seen as a normal consequence of aging, and the underlying cause may not even be treated.^[46]^ Further study has shown that the most prevalent type of anemia in the elderly is anemia of chronic disease (ACD).^[45]^ Likewise, as mild anemia was the most prevalent anemia in this study, it is important to look into any potential causes, including the young age group. Overall, 49% of the anemia in the survey was classified as moderate or severe. These types of anemia frequently occur in young people aged 15 to 30,^[44]^ This has an impact on society’s productive age group. Since they have a considerably greater impact on patients than the mild form of anemia, these categories of anemic patients deserve attention.

In addition, the relationship between PLT and RBCs contributes to our understanding of the pathophysiology of inflammatory diseases, particularly rheumatoid arthritis, which can result in anemia of inflammation. In an in vitro study, red blood cell-derived microparticles (RMPs) were able to induce PLT hyper-stimulation after collagen activation.^[47]^ Recently confirmed to induce in vivo PLT-PLT aggregates, these RMPs. Direct contact between PLT and RBC has also been found to involve certain adhesion proteins. As a member of the glycoprotein family, adhesion molecule 4 (also known as CD-242 or ICAM-4) on the membranes of RBCs may be able to directly bind to integrin IIbb3 of PLT, indicating the direct impact of RBCs on the activation of thrombotic and inflammatory.^[48]^ It is important to note that hemoglobin measurements were adjusted for altitude as recommended by WHO, which suggests a correction factor of 1.3 for readings taken at an average altitude of 2700 meters. This adjustment is crucial in accurately diagnosing anemia in high altitude populations, as altitude can significantly affect hemoglobin levels by reducing oxygen availability, prompting the body to increase red cell production to compensate. This physiological response can lead to higher hemoglobin levels in individuals living at altitude, necessitating correction factors to avoid misdiagnosis of anemia. The limitation of the study was as follows: The study included only 176 anemic patients which affects the generalizability of the results. Additionally, while the study focused on correlating RBC parameters with platelet counts, it did not assess potential confounding factors which could influence anemia. Finally, the reliance on a cross-sectional design restricts the ability to establish causal relationship and the lack of advanced statistical methods further limits the comprehensiveness of the analysis. Therefore, we recommend that further studies to consider the evaluation of iron deficiencies anemia indicators like serum ferritin, and serum transferrin, as well as serum folate and serum Vit-B12. Longitudinal studies should also be conducted to assess potential confounding factors.

## 5. Conclusions

PLT count was significantly, positively correlated with RBC, and negatively correlated with MCV, MCH, and MCHC (*r* (*P*) = 0.168 (.026) and −0.252 (.001), −0.275 (.001), −0.218 (.004) respectively) in anemic patients. It was also negatively correlated with Hgb and Hct levels in the healthy control (*r* (*P*) = −0.266 (.001) and −0.149 (.049), respectively, and positively correlated with RDW. The red blood cell parameters and platelet count in anemic patients are important to identify and understand their association with clinical implications. Such correlation will enable physicians to make diagnoses and administer treatments.

## Acknowledgments

The authors thank the staff and study participants for their important contributions.

## Author contributions

**Conceptualization:** Mohammedamin Jundi, Edosa Tadasa, Wondimagegn Adissu.

**Formal analysis:** Mohammedamin Jundi, Edosa Tadasa, Wondimagegn Adissu.

**Investigation:** Mohammedamin Jundi.

**Resources:** Mohammedamin Jundi, Edosa Tadasa, Wondimagegn Adissu.

**Software:** Mohammedamin Jundi.

**Supervision:** Edosa Tadasa, Wondimagegn Adissu.

**Validation:** Edosa Tadasa, Wondimagegn Adissu.

**Writing – original draft:** Mohammedamin Jundi.

**Writing – review & editing:** Edosa Tadasa.

## References

[1] Chan M; WHO. Haemoglobin concentrations for the diagnosis of anemia and assessment of severity. Geneva, Switzerland: World Health Organization. 2011:1–6.

[2] Bekele A Tilahun M Mekuria A. Prevalence of anemia and its associated factors among pregnant women attending antenatal care in health institutions of Arba Minch Town, Gamo Gofa Zone, Ethiopia: a cross-sectional study. Hindawi Publishing Corporation, Anemia. 2016;2016:1073192 10.1155/2016/1073192 PMC4779815 27022481

[3] Mehta AB Hoffbrand AV. Hematology at a Glance. 4th Edition. London: John Wiley & Sons, Ltd Registered; 2014:1–137.

[4] Kalairajan S. A study of red cell distribution width and RBC indices in iron deficiency anemia. Asian J Med Res. 2019;8:ME11–4.

[5] Elmardi KA Adam I Malik EM. Prevalence and determinants of anemia in women of reproductive age in Sudan: analysis of a cross-sectional household survey. BMC Public Health. 2020;20:1–12. 32680488 10.1186/s12889-020-09252-w PMC7367227

[6] Sherawat M Mittal V Arora S Kumar R. Patterns of anemia in elderly patients in relation with RBC indices. Int J Curr Res Rev. 2021;13:78–82.

[7] Tadesse AW Hemler EC Andersen C. Anemia prevalence and etiology among women, men, and children in Ethiopia: a study protocol for a national population-based survey. BMC Public Health. 2019;19:1–8. 31651278 10.1186/s12889-019-7647-7 PMC6814127

[8] Chulilla JAM Colás MSR Martín MG. Classification of anemia for gastroenterologists. World J Gastroenterol. 2009;15:4627–37. 19787825 10.3748/wjg.15.4627 PMC2754510

[9] Greer JP. Wintrobe s Clinical Hematology. 13th Edition. In: Greer JP Arber DA, eds. China: Lippincott Williams & Wilkins; 2014:2306.

[10] Adamu AL Crampin A Kayuni N. Prevalence and risk factors for anemia severity and type in Malawian men and women: Urban and rural differences. Popul Health Metr. 2017;15:12. 28356159 10.1186/s12963-017-0128-2 PMC5371260

[11] Alvarez-Uria G Naik PK Midde M Yalla PS Pakam R. Prevalence and severity of anemia stratified by age and gender in rural India. Anemia. 2014;2014:176182. 25614831 10.1155/2014/176182 PMC4277798

[12] Periayah MH Halim AS Mat Saad AZ. Mechanism action of platelets and crucial blood coagulation pathways in hemostasis. Int J Hematol Oncol Stem Cell Res. 2017;11:319–27. 29340130 PMC5767294

[13] Kumar D Kasukurti P Murthy S. Erythrocytes and platelets: a critical analysis of their ontogenic relationship through automated parameters. J Clin Diagn Res. 2017;11:EC05–BC08. 10.7860/JCDR/2017/25153.9807 PMC5483665 28658763

[14] Rajagopal L Raja V Abdullah SM Arunachalam S Ganapathy S. Original article correlative analysis of red blood cell and platelet parameters predicts the risk of thrombosis in patients with iron deficiency anemia. National Journal of Basic Medical Sciences. 2015;9:16–26.

[15] Demetz G Laux M Scherhag A. The influence of erythropoietin on platelet activation, thrombin generation and FVII/active FVII in patients with AMI. Thrombosis J. 2014;12:1–7. 10.1186/1477-9560-12-18 PMC4165375 25228850

[16] Samanta P Senapati L. Association of nutritional anemia with leukocyte and platelet counts in people of Odisha. Natl J Physiol Pharm Pharmacol. 2017;8:1.

[17] Kloub MN Yassin MA. Oral iron therapy-induced neutropenia in patient with iron deficiency anemia. Case Rep Oncol. 2020;13:721–4. 32774264 10.1159/000507730 PMC7383194

[18] Park MJ Park PW Seo YH. The relationship between iron parameters and platelet parameters in women with iron deficiency anemia and thrombocytosis. Platelets. 2013;24:348–51. 22738419 10.3109/09537104.2012.699641

[19] Chalise S Acharya N Pradhan SB. Correlation between iron parameters and platelet parameters in iron deficiency anemia. J Inst Med Nepal. 2019;41:35–8.

[20] Özdemir N Celkan T Kebudi R Bor M Yildiz I. Demir eksikliǧi anemisi ve demir tedavisi ile ilişkili sitopeni: İki olgu sunum. Turk J Hematol. 2011;28:243–4. 10.5152/tjh.2011.65 27264378

[21] Samsuria IK Judiono Watuguly TW. Correlation between thrombocyte, erythrocyte, and ratio thrombocyte leucocyte in a patient with cardiovascular disease. Bali Med J. 2021;10:433–6.

[22] Fahim FM Helal SR Fathalla E Saeed AN Galal SM. Platelet abnormalities in children with iron deficiency anemia. J Curr Med Res Pract. 2022;7:22.

[23] Ray S Chandra J Sharma S. Clinico-hematological study of abnormalities of platelet count in children with iron deficiency anemia. Int J Contemp Pediatr. 2019;6:1519–23.

[24] Samia Z. Population and housing census 2007: oromia statistical. 2007. http://ndl.ethernet.edu.et/browsetype=author&value=Samia+Zekaria.

[25] Kang H. Sample size determination and power analysis using the G*Power software. J Educ Eval Health Prof. 2021;18:17. 34325496 10.3352/jeehp.2021.18.17 PMC8441096

[26] Alsaeed AH. An analysis of hematological parameters to assess the prevalence of anemia in elderly subjects from Saudi Arabia. Genet Test Mol Biomarkers. 2011;15:697–700. 21574853 10.1089/gtmb.2011.0030

[27] Haile K Yemane T Tesfaye G Wolde D Timerga A Haile A. Anemia and its association with helicobacter pylori infection among adult dyspeptic patients attending Wachemo University Nigist Eleni Mohammad Memorial Referral Hospital, Southwest Ethiopia: a cross-sectional study. PLoS One. 2021;16:e0245168. 33444345 10.1371/journal.pone.0245168 PMC7808578

[28] WHO. Weekly iron and folic acid supplementation as an anaemia-prevention strategy in women and adolescent girls: lessons learnt from implementation of programmes among nonpregnant women of reproductive age. Geneva: World Health Organization; 2018:40.

[29] Kumar V Choudhry VP. Iron deficiency and infection. Indian J Pediatr. 2010;77:789–93. 20589461 10.1007/s12098-010-0120-3

[30] Evstatiev R Bukaty A Jimenez K. Iron deficiency alters megakaryopoiesis and platelet phenotype independent of thrombopoietin. Am J Hematol. 2014;89:524–9. 24464533 10.1002/ajh.23682 PMC4114532

[31] Kühnl A Gökbuget NSA. Platelets & thrombopoiesis. J Clin Lab Anal. 2010;308:34.

[32] Raslova H Kauffmann A Sekkaï D. Interrelation between polyploidization and megakaryocyte differentiation: a gene profiling approach. Blood. 2007;109:3225–34. 17170127 10.1182/blood-2006-07-037838

[33] Evstatiev R Gasche C. Iron sensing and signaling. Gut. 2012;61:933–52. 22016365 10.1136/gut.2010.214312

[34] Kuku I Kaya E Yologlu S Gokdeniz R Baydin A. Platelet counts in adults with iron deficiency anemia. Platelets. 2009;20:401–5. 19658005 10.1080/09537100903137306

[35] Morris VK Spraker HL Howard SC Ware RE Reiss UM. Severe thrombocytopenia with iron deficiency anemia. Pediatr Hematol Oncol. 2010;27:413–9. 20670168 10.3109/08880011003739455 PMC3439835

[36] Rammohan A Awofeso N Robitaille M-C. Addressing female iron-deficiency anaemia in India: is vegetarianism the major obstacle? ISRN Public Health. 2012;2012:1–8.

[37] Upadhyaya Kafle S Singh M Kafle N Sinha A. Hemogram components and platelets count variation in anemic patients attending Birat Medical College and Teaching Hospital, Morang, Nepal. J Pathol Nepal. 2021;11:1825–9.

[38] Berad A Gurbani N. To study the relation between hemoglobin level and platelet count. Int J Res Med Sci. 2016;4:4759–61.

[39] Jadhav SU Khaparde S. Study of the red cell indices, hemogram and platelet variations in anemic (<10% gm) patients by automatic cell counter in a tertiary care center, Ahmednagar, Maharashtra, India. Int J Res Med Sci. 2017;5:1582.

[40] Tettamanti M Lucca U Gandini F. Prevalence, incidence, and types of mild anemia in the elderly: the “Health and Anemia” population-based study. Haematologica. 2010;95:1849–56. 20534701 10.3324/haematol.2010.023101 PMC2966906

[41] Callera F Callera AF Da Silva AM Rosa ES. Prevalence of anemia in a sample of elderly southeastern Brazilians. Rev Bras Hematol Hemoter. 2014;37:43–7. 25638767 10.1016/j.bjhh.2014.06.005 PMC4318851

[42] Sgnaolin V Engroff P Ely LS. Hematological parameters and prevalence of anemia among free-living elderly in south Brazil. Rev Bras Hematol Hemoter. 2013;35:115–8. 23741189 10.5581/1516-8484.20130032 PMC3672121

[43] Guralnik JM Ershler WB Schrier SL Picozzi VJ. Anemia in the elderly: a public health crisis in hematology. Hematology. 2005;10:528–32. 10.1182/asheducation-2005.1.528 16304431

[44] Aslam A Tauseef A Zahra Q Arshad F Sajjad S. Prevalence of major types of anemia among adults in local population of Lahore. Indo Am J P Sci. 2019;6:13430–4.

[45] Gaskell H Derry S Andrew Moore R McQuay HJ. Prevalence of anemia in older persons: systematic review. BMC Geriatr. 2008;8:1–8. 18194534 10.1186/1471-2318-8-1 PMC2248585

[46] Benjamin H. Chen MCCF and SGHBs. 059. Vol. 59. 2004.

[47] Xue L Tao L Sun H. Association between blood PLT and RBC related indices and disease activity in patients with rheumatoid arthritis. Int J Gen Med. 2022;15:573–81. 35046715 10.2147/IJGM.S351505 PMC8763267

[48] Du VX Huskens D Maas C Al Dieri R De Groot PG De Laat B. New insights into the role of erythrocytes in thrombus formation. Semin Thromb Hemost. 2014;40:72–80. 24356930 10.1055/s-0033-1363470

